# Comparative genomics of *Flavobacterium columnare* unveils novel insights in virulence and antimicrobial resistance mechanisms

**DOI:** 10.1186/s13567-021-00899-w

**Published:** 2021-02-12

**Authors:** Annelies Maria Declercq, Laurentijn Tilleman, Yannick Gansemans, Chloë De Witte, Freddy Haesebrouck, Filip Van Nieuwerburgh, Annemieke Smet, Annemie Decostere

**Affiliations:** 1 Department of Pathology, Bacteriology and Avian Diseases, Faculty of Veterinary Medicine, Ghent University, Salisburylaan 133, 9820 Merelbeke, Belgium; 2 Flanders Marine Institute, Wandelaarkaai 7, 8400 Ostend, Belgium; 3 Laboratory of Aquaculture & Artemia Reference Center, Department of Animal Sciences and Aquatic Ecology, Faculty of Bioscience Engineering, Ghent University, Coupure Links 653, 9000 Ghent, Belgium; 4 Laboratory of Pharmaceutical Biotechnology, Department of Pharmaceutics, Faculty of Pharmaceutical Sciences, Ghent University, Ottergemsesteenweg 460, 9000 Ghent, Belgium; 5 Laboratory Experimental Medicine and Pediatrics, Faculty of Medicine and Health Sciences, University of Antwerp, Universiteitsplein 1, 2610 Wilrijk, Belgium

**Keywords:** *Flavobacterium columnare*, Genome comparison, Virulence, Antimicrobial resistance

## Abstract

This study reports the comparative analyses of four *Flavobacterium columnare* isolates that have different virulence and antimicrobial resistance patterns. The main research goal was to reveal new insights into possible virulence genes by comparing the genomes of bacterial isolates that could induce tissue damage and mortality versus the genome of a non-virulent isolate. The results indicated that only the genomes of the virulent isolates possessed unique genes encoding amongst others a methyl-accepting chemotaxis protein possibly involved in the initial colonization of tissue, and several VgrG proteins engaged in interbacterial competition. Furthermore, comparisons of genes unique for the genomes of the highly virulent (HV) carp and trout isolates versus the, respectively, low and non-virulent carp and trout isolates were performed. An important part of the identified unique virulence genes of the HV-trout isolate was located in one particular gene region identified as a genomic island. This region contained *ara*C and *nod*T genes, both linked to pathogenic and multidrug-resistance, and a *lux*R-gene, functional in bacterial cell-to-cell communication. Furthermore, the genome of the HV-trout isolate possessed unique sugar-transferases possibly important in bacterial adhesion. The second research goal was to obtain insights into the genetic basis of acquired antimicrobial resistance. Several point-mutations were discovered in gyrase-genes of an isolate showing phenotypic resistance towards first and second-generation quinolones, which were absent in isolates susceptible to quinolones. Tetracycline-resistance gene *tet*A was found in an isolate displaying acquired phenotypic resistance towards oxytetracycline. Although not localized on a prophage, several flanking genes were indicative of the gene’s mobile character.

## Introduction

*Flavobacterium columnare* (*F. columnare*) is a Gram-negative, bacterial, freshwater fish pathogen that causes columnaris disease. The latter is characterized by gill, fin, and skin lesions [1, 2]. *F. columnare* belongs to the family *Flavobacteriaceae* [1], which is part of the phylum Bacteroidetes. The *F. columnare* cluster is subdivided into five genomovars: I, I/II, II, II-B and III [3].

*Flavobacterium columnare* is distributed globally and may infect many different cultured and wild freshwater fish species, including carp, catla, channel catfish, eel, goldfish, perch, salmonids, and tilapia [1, 2]. Tropical freshwater aquarium fish may also be affected [4]. In channel catfish (*Ictalurus punctatus*), *F.columnare* is in the top‐three diseases reported to cause morbidity and mortality resulting in yearly losses of millions of dollars [5]. The prevalence of columnaris disease in cyprinids is also noteworthy. Carp (*Cyprinus carpio*), for example, are amongst the world’s most-produced food fish [6, 7] and koi carp, in particular, have high economic value as ornamental fish species [8, 9].

Various reports have described differences in virulence among *F. columnare* strains [1, 10–14]. Colonization of the host tissue by *F. columnare* plays a crucial part in this. This process is initiated by the chemotaxis of the bacteria to the host tissues. Former studies on *F. columnare* isolates of channel catfish revealed that virulent isolates have an increased chemotactic response towards channel catfish mucus [13, 15], suggesting that chemotaxis could be associated with virulence. Bader et al. [16] reported that *F. columnare* cells were attached to the skin and gill mucus of channel catfish as early as 5 min after a bath immersion challenge. Similar results were previously obtained in carp and rainbow trout (*Oncorhynchus mykiss*) [13]. The latter revealed that virulent *F. columnare* carp and trout isolates attached faster and in higher numbers to the gill tissue, which eventually led to the mortality of the host. This mortality rate was not seen for fish exposed to a non-virulent isolate as in those, no tissue colonization by the bacterial cells was displayed [10, 11]. Sugars would also play an important role in the adhesion of *F. columnare* to the host tissue. Decostere et al. [17], for example, reported that treatment of either *F. columnare* bacterial cells or gill tissue with sodium metaperiodate (cleaves the C–C bond between vicinal hydroxyl groups of sugar) or with various sugars significantly reduced bacterial adhesion. This lead to the hypothesis that the interaction between a bacterial lectin and its host’s carbohydrate receptor explained the diminished adhesion [17].

Once adhered to the host tissue, bacterial cell-to-cell communication, a process known as quorum sensing, allows planktonic cells to switch to biofilm formation [18]. In this mechanism of gene regulation, bacteria coordinate the expression of certain genes in response to the concentration of small signal molecules. The exact mechanisms of bacterial cell-to-cell communication in *F. columnare* is currently unknown. However, in other Gram-negative bacteria, the involvement of two important proteins in the regulation of QS has been described [19]. An I-protein is the enzyme that synthesizes the signalling molecule [19, 20]. The signal molecules are detected by an R-protein, a transcriptional regulator. The signal molecules accumulate extracellularly and once a certain threshold concentration has been reached, they bind to a response regulator (LuxR homolog). Subsequently, the LuxR/signal molecule complex activates the transcription of QS-regulated genes [19, 21].

In the past years, the number of *Flavobacterium* genomes reported has increased. For *F. columnare*, some comparative genomic studies are available [22–24]. However, these studies focused on the genomes of different catfish and tilapia species, particularly on isolates that were retrieved from diseased fish only. In this study, we performed comparative analyses of four *F. columnare* strains, including two strains isolated from trout (*Oncorhynchus mykiss*), and two from carp (*Cyprinus carpio*). These isolates belong to genomovar I and possess different virulence patterns [10, 11]. These moreover show differences in antimicrobial susceptibility [25]. The comparative genomics approach conducted in this research is the first to focus on identifying virulence genes based on the comparison between *F. columnare* isolates with a different virulence and antimicrobial susceptibility pattern in carp and trout.

## Materials and methods

### *Flavobacterium* species, library preparation and whole-genome sequencing

Four *F. columnare* isolates belonging to genomovar I [25] were included in this study for genome sequencing. The virulence pattern and antimicrobial resistance profile of all isolates, as previously described [10, 11, 25], are shown in Table 1. Briefly, the two strains that could induce tissue colonization and cause 100% mortality within 24 h after the onset of an immersion challenge, were assigned highly virulent (HV), which is the case for, respectively, the carp and trout isolates 04017018 and JIP P11/91 [10, 11]. The carp isolate CDI-A was assigned low virulent (LV), as it could induce tissue colonization leading to mortality, but in up to ten percent of fish challenged [10, 11]. The trout isolate ATCC49512 was not able to adhere to the host tissue nor to induce mortality, and was hence labelled as non virulent (NV) [10, 11]. The genome sequence of isolate *F. columnare* ATCC 49512 is publicly available from the NCBI database [26]. The genomes of the other three strains were sequenced and assembled during this study. Briefly, the *F. columnare* strains were cultivated as described before [25] and whole DNA was extracted using the Qiagen (Venlo, The Netherlands) Blood & Tissue kit, following the manufacturer’s instructions. The qualities and quantities of the extracted DNA were evaluated using a NanoDrop spectrophotometer (NanoDrop™, Themo Fisher Scientific, Merelbeke, Belgium) and included for further library preparation and subsequent sequencing. *F. columnare* genomic DNA was then sheared on a Covaris S2 sonicator (Covaris, Woburn, Massachusetts, USA) aiming for 800 bp fragments. The fragmented DNA was used to build a sequencing library with the NEBNext Ultra DNA Library Prep Kit for Illumina (New England Biolabs, Ipswich, Massachusetts, USA). The sequencing library was size-selected on a 2% agarose E-gel. Fragments ranging from 600 to 900 bp were cut out of the gel and purified with the Zymoclean Gel Recovery Kit (Zymo Research, Irvine, California, USA). The isolated library fragments were submitted to 8 PCR cycles and recovered using AMPure XP magnetic beads (Beckman Coulter, Indianapolis, Indiana, USA). Library quality was checked on an Agilent Bioanalyzer using a High Sensitivity Chip (Agilent Technologies, Santa Clara, California, USA) and concentration was measured via qPCR according to the qPCR Quantification Protocol (Illumina, San Diego, California, USA). Sequencing was done as paired-end 300 on a MiSeq (Illumina).

**Table 1:** ***Flavobacterium* strains included in this study, their GenBank-accession numbers, isolation sources, and antimicrobial resistance information**

*F. columnare* isolate	Accession number	Isolation source	Virulence profile^b^	Antimicrobial resistance—MIC-values^c^
*Flavobacterium columnare* ATCC_49512	GCA_000240075.2	Trout (*Oncorhynchus mykiss*), genomovar I, France	Non-virulent (NV)	No antimicrobial resistance detected
*Flavobacterium columnare* JIP P11/91^a^	GCA_015499905.1	Trout, genomovar I, France	Highly virulent (HV)	No antimicrobial resistance detected
*Flavobacterium columnare* 04017018^a^	GCA_015499955.1	Diseased koi carp (*Cyprinus carpio*), genomovar I, The Netherlands	HV	Oxytetracycline—8 μg/mL
*Flavobacterium columnare* CDI-A^a^	GCA_015499935.1	Koi carp, genomovar I, The Netherlands	Low virulent (LV)	Enrofloxacin—1 μg/mL, flumequin—8 μg/mL, oxolinic acid—12 μg/mL

^a^Indicates the isolates for which whole-genome sequencing was performed in this study.

^b^Results from Declercq et al. [10, 11].

^c^Results from Declercq et al. [25].

### Genome assembly and annotation

Removal of residual adapters and quality trimming of raw sequencing reads was done with Cutadapt [27], version 1.15, using a phred cut-off of 20 and removal of reads containing ambiguous nucleotides or having a final length shorter than 100 nucleotides. The quality of the reads was checked using FastQC [28], version 0.11.5. The trimmed paired-end reads were assembled into genome scaffolds with SPAdes [29], version 3.11.1, using options—careful and—k 21,33,55,77,99,127. Scaffolds smaller than 2000 nucleotides were discarded. The quality of the obtained genome assemblies was checked with QUAST [30], version 5.0.0, and BUSCO [31] version 3.0.2. Raw sequencing reads and the assembled genomes of the three isolates were submitted at NCBI (BioProject accession PRJNA431138) and are publicly available [32]. The GenBank accession numbers of the de novo assembled genomes are shown in Table 1. All new genomes, as well as the genome of strain ATCC 49512, were subjected to gene finding and automatic annotation using RAST (Rapid Annotations using Subsystems Technology) version 2.0 and SEED viewer [33].

### Comparative analysis of *F. columnare* isolates

The pan-genome ortholog clustering tool (PanOCT) was applied for a pan-genomic analysis of the four isolates [34]. This tool looked for gene differences between these four isolates. Gene locations are formatted as NODEn:s-e where n is a unique scaffold number, s and e are the gene’s start and end position respectively. Scaffold names are based on their original names as generated by the de novo genome assembly software. The publicly available submitted genomes contain newly assigned scaffold names by NCBI, as well as the original name (formatted as NODE_n). To identify possible virulence factors encoded in *F. columnare*, the virulence factor database (VFDB) was downloaded [35, 36], and a local BLAST search was conducted using BLAST + version 2.2.31. To identify phage elements in the three newly sequenced *F. columnare* genomes, sequences were submitted to the PHAge Search Tool- Enhanced Release (PHASTER) server [37]. Additionally, the presence of genes involved in *Flavobacterium* secretion systems was investigated. MacSyFinder 1.0.5 and TXSScan were applied to detect protein secretion systems and their appendages in the genomes [38, 39].

Variant detection in the gyrase genes of isolates CDI-A and 04017018 was performed using CLC Genomics Workbench against those of *F. columnare* reference CP0003222.2. Jalview 2.11.1 [40] was used to verify whether these SNPs present in isolate CDI-A were associated with amino acid substitutions. Then, to investigate the impact of these amino acid substitutions on gyrase activity, the online PredictSNP software was used [41]. PredictSNP, MAPP, PhD-SNP, PolyPhen-1 and -2, SIFT, SNAP, nsSNPAna-lyzer, and PANTHER were run with default parameters. Using the online I-Mutant Suite 3.0 software [42], the impact of amino acid substitutions on gyrase stability was investigated. Finally, ConSurf Server [43] was run with default parameters to investigate if the amino acid substitutions were located in conserved regions.

## Results

The four genomes were assembled at scaffold level and are therefore incomplete. However, their sizes (3.09 to 3.21 Mbp) are within the range of the complete genomes for this species as listed by NCBI (3.03 to 3.27 Mbp). The GC content (31.2 to 31.5%) and number of protein coding genes (2626 to 2770) was similar for the assembled genomes (Table 2). Amino acid composition (Additional file 1A) and predicted protein lengths (Additional file 1B) were similar between the four *F. columnare* genomes. Predicted subcellular localization of proteins was also similar (Additional file 1C).

**Table 2:** **Chromosome features of** **Flavobacterium columnare- strains sequenced in this study** ^a^** compared to the genomovar-I type strain**

Chromosome feature	*F. columnare* ATCC 49512	*F. columnare* 04017018^a^ (S4)	F. columnare CDI-A^a^ (S5)	*F. columnare* JIP P11/91^a^ (S6)
Genome size	3 162 432	3 092 581	3 138 432	3 216 153
G+C content (%)	31.5	31.2	31.4	31.4
Coverage	NA	231	261	198
Sum of coding and non-coding genes	2793	2771	2815	2907
Number of contigs/scaffolds	1	55	60	104
Protein coding genes	2626	2632	2680	2770
Average gene length (bp)	993	977	976	966
Plasmid	No	No	No	No

^a^Indicates the isolates for which whole-genome sequencing was performed in this study.

The SEED viewer “sequence based comparison” tool allowed the virulent *F. columnare* isolates to be aligned to the reference genome and each other. The cut-off value for homologues was set at 70% protein sequence identity (i.e. BLAST e-cutoff value). This resulted in a list of genes being present in a reference genome in chromosomal order and displayed hits on the comparison organisms accordingly. This allowed determining 156 protein-encoding genes, of which 114 encoded for hypothetical proteins, that were present in the genomes of the three virulent isolates, while being absent in the NV *F. columnare* trout isolate. The protein-encoding genes with a known function as annotated by RAST are listed in Additional file 2. These genes were then compared to the VFDB, to look for those associated with virulence. The resulting protein encoding genes are marked in bold in Additional file 2 and include several transferases, membrane proteins, several VgrG proteins and a methyl-accepting chemotaxis protein (MCP).

**Figure 1:**
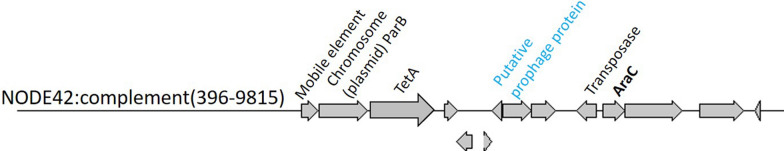
**Schematic representation of unique genes on NODE42 in carp isolate 04017018.** This region identified in this highly virulent carp isolate 04017018 possesses unique genes (black) when compared to the genome of low virulent trout isolate CDI-A. Genes marked in bold are also predicted to be involved in virulence when identifying them via Virulence Factor Database. Hypothetical ones are not labelled.

Furthermore, the SEED viewer tool allowed to align the genomes of the HV *F. columnare* carp and trout isolates to those of the, respectively low (carp) and non-virulent (trout) isolates. For the comparison of the *F. columnare* carp genomes, 80 genes (of which 62 hypothetical) were present only in the genome of the HV carp isolate while absent in the LV one. Regarding virulence, the homologous virulence-associated proteins encoded for the HV carp isolate genome include a transcriptional regulator *ara*C [NODE42:complement (3102–3539)] and a glycosyltransferase involved in lipopolysaccharide biosynthesis (LPS) [NODE6:complement (123808–124008)]. An overview of the unique protein-encoding genes (excluding the hypothetical ones) in the HV carp isolate genome, including the ones predicted to be involved in virulence when identifying them via the VFDB, are presented in Additional file 3. The HV carp isolate also showed acquired resistance against oxytetracycline as determined before [25]. The gene encoding the AraC family protein in the HV carp isolate genome is flanked by a transposase, a putative prophage element, and a gene coding for TetA antimicrobial resistance protein, as shown in Figure 1.

**Figure 2:**
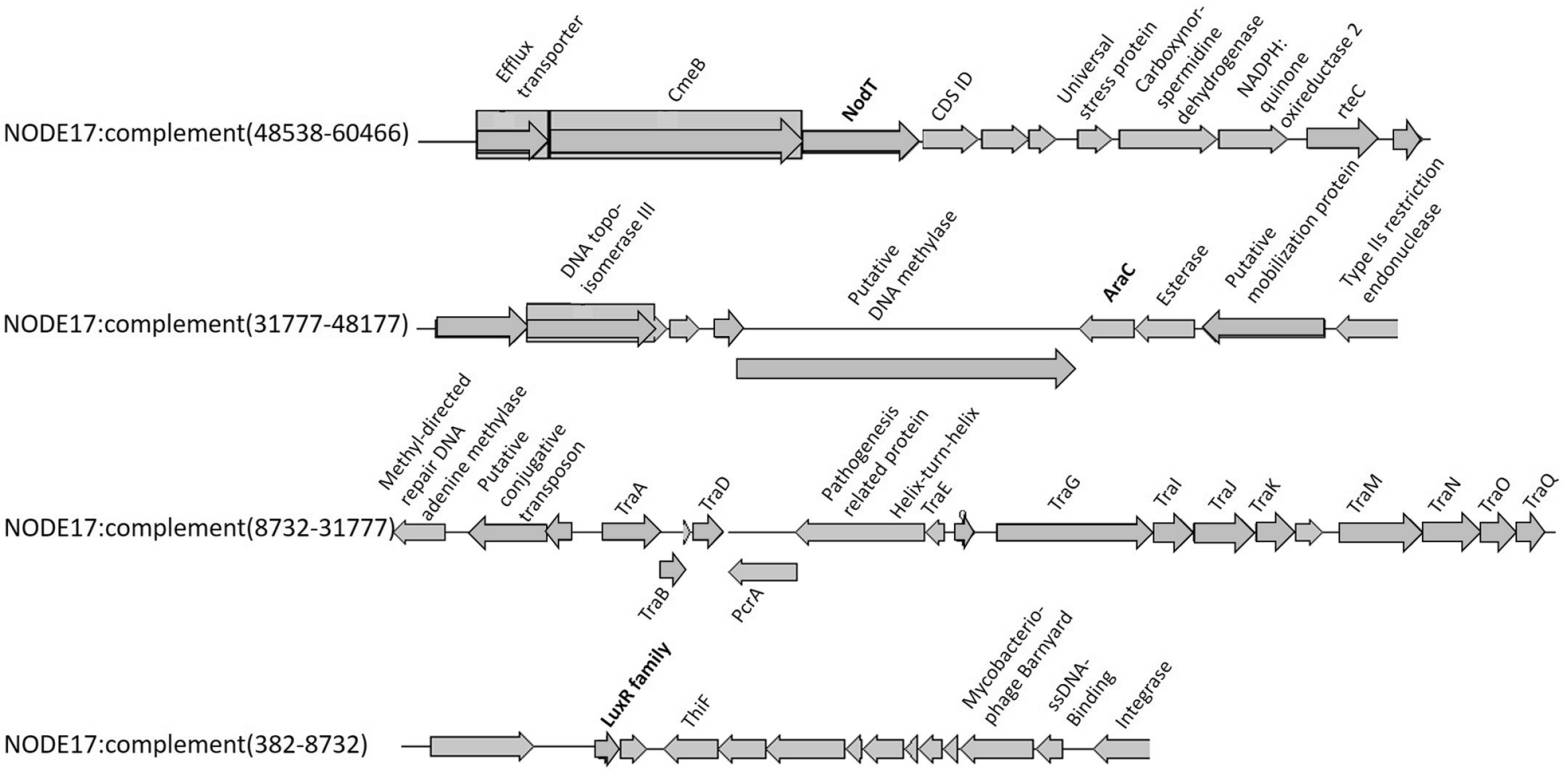
**Schematic representations of unique genes grouped on NODE17 (genomic island) in trout isolate JIPP11/91.** When comparing the genome of this highly virulent trout isolate with the one from the non-virulent trout isolate ATCC 49512, several unique genes, present only in the highly virulent isolate are grouped on NODE17, which is recognized as a genomic island by the IslandPaht-DIMOB and SIGI-HMM method. Several of these unique genes are also predicted to be involved in virulence when identifying them via Virulence Factor Database. These are marked in bold. The hypothetical ones are not labelled.

For trout, 374 genes (of which 263 hypothetical ones) were present only in the HV trout and absent in NV trout isolate. Next to the genes reported in Additional file 2, genes involved in transposable elements, transcription, antimicrobial resistance, universal stress and ulcer associated adenine specific DNA methyltransferase were identified. Regarding virulence, the homologous virulence-associated proteins encoded for the HV trout isolate genome include a gene encoding for a membrane protein (NODE11:22223–23335), *nod*T [NODE17:complement (54957–56384), *ara*C (NODE17:36752–37645), *lux*R (NODE17:complement (8305–8706)], and a unique *vgr*G [NODE47:complement(5031–5630)]. An overview of the unique protein-encoding genes (excluding the hypothetical ones) in the HV trout isolate genome, including the ones predicted to be involved in virulence when identifying them via the VFDB, are presented in Additional file 4. For the HV *F. columnare* strain isolated from trout, some very interesting virulence genes are grouped on NODE17, which is recognized as a genomic island by the IslandPaht-DIMOB and SIGI-HMM method. The latter is represented in Figure 2.

MacSyFinder and TXSScan could detect multiple genes encoding for protein members of several secretion systems. Most of the systems are positioned on different scaffolds but are grouped within a scaffold. The four genomes carry a complete T1SS, consisting of three essential components: ABC-transporter (represented by gene *abc*), porin (*omf*), and the inner membrane-anchored adaptor protein (*mfp*). Furthermore, the T9SS carries genes involved in gliding (*gld*J, *gld*K, *gld*L, *gld*M, *gld*N), spreading (*spr*A, *spr*E, and *spr*T) and porphyrin accumulation on the cell surface (*por*O, *por*U, *por*V). When observing the T4P, Pil B, C, M, and Q were identified in all sequenced *F. columnare* isolates. All four genomes seem to possess *sct*N in the T3SS, *tss*H in the T6SSi and a complete T6SSiii. In T4SS type B, several differences were identified. Apart from *t4cp1* in the HV carp isolate, no genes were identified in the T4SS typeB for isolates 04017018 and ATCC49512. No other differences could be encountered. Table 3 gives a comparative overview of the secretion systems.

**Table 3:** **Secretion systems distribution in the four** ***Flavobacterium columnare* genomes**

	T2SS														
	M.G									A.G				F.G	
	gspD	gspG	gspH	gspM	gspE	gspJ	gspK	gspF	gspI	gspL	gspO	gspC	gspN	pilM	tadZ
ATCC 49512						1								1	
JIP P11/91^a^						1								1	
04017018^a^						1								1	
CDI-A^a^						1								1	

	T4P												
	M.G									A.G	F.G		
	pilM	pilI_pilV	pilC	pilAE	pilP	pilO	pilQ	pilT_pilU	pilB	pilD	gspC	tadZ	gspN
ATCC 49512	1		1				1		1				
JIP P11/91^a^	1		1				1		1				
04017018^a^	1		1				1		1				
CDI-A^a^	1		1				1		1				

	T3SS												T1SS		
	M.G.									F.G.			M.G.		
	sctN	sctJ	sctT	sctV	sctC	sctU	sctQ	sctR	sctS	fliE	flgB	flgC	mfp	abc	omf
ATCC 49512	2												3	6	4
JIP P11/91^a^	2												3	6	5
04017018^a^	2												3	6	4
CDI-A^a^	2												3	6	5

	T6SSi													
	M.G.													
	tssL	tssI	tssJ	tssH	tssD	tssB	tssM	tssC	evpJ	tssG	tssE	tssK	tssF	tssA
ATCC 49512				2										
JIP P11/91^a^				2										
04017018^a^				2										
CDI-A^a^				2										

	T6SSiii											
	M.G.											
	tssK	tssC	tssE	tssI	tssN	tssP	tssH	tssF	tssD	tssG	tssB	tssQ
ATCC 49512	1	1	1	4	1	1	1	1	8	1	1	
JIP P11/91^a^	1	1	1	1	1	1	1	1	2	1	1	
04017018^a^	1	1	1	1	1	1	1	1	4	1	1	
CDI-A^a^	1	1	1	1	1	1	1	1	4	1	1	

	T4SS type B														
	M.G.			A.G.											
	t4cp1	MOBB	virb4	traF	traN	traO	traJ	traL	traP	traI	traH	traK	traQ	traE	traM
ATCC 49512															
JIP P11/91^a^	2	1	1	1	1	1	1			1		1	1	1	1
04017018^a^	1														
CDI-A^a^	2	1	1	1	1	1	1			1		1	1	1	1

	T9SS										
	M.G.								A.G.		
	gldM	sprT	gldN	sprA	porV	gldL	sprE	gldK	porQ	porU	gldJ
ATCC 49512	1	1	1	1	1	1	1	1	2	1	1
JIP P11/91^a^	1	1	1	1	1	1	1	1	2	1	1
04017018^a^	1	1	1	1	1	1	1	1	2	1	1
CDI-A^a^	1	1	1	1	1	1	1	1	2	1	1

^a^ Indicate the isolates for which whole-genome sequencing was performed in this study.

^b^ Forbidden genes are based on Abby et al. [39].

M.G., mandatory genes (genes that must be present in the genome to define this system); A.G., accessory genes (genes that can be present, but do not have to be found in every case); and F.G., forbidden genes^b^ (genes that must not be present in the system).

When searching for known point mutations associated with fluoroquinolone resistance in the LV carp isolate CDI-A, 11 SNPs were detected in several gyrase genes upon comparing those with the ones from the reference genome of *F. columnare*, the latter not displaying resistance towards fluoroquinolones [25]. These SNPs were present in *gyr*A (T to G, position 244), two single point mutations in Topoisomerase IV subunit A (G to T, position 1221; T to G position 1221), three-point mutations in Topoisomerase IV subunit B (G to A, position 154; T to G, position 1779; A to G, position 1782), and five-point mutations in DNA topoisomerase I (A to G, position 273; G to A position 405; G to A, position 468; G to A, position 617; A to G, position 2151) (Additional file 5). However, seven of these point mutations were also present in the genome of the HV carp isolate, which also did not reveal resistance towards any of the quinolone antimicrobial agents. The four unique SNPs which were present in isolate CDI-A, were the one in *gyr*A (T to G, position 244), and three in DNA topoisomerase I (A to G, position 273; G to A position 405; G to A, position 468) (Additional file 5). Using Jalview, only the SNP present in *gyr*A resulted in an amino acid substitution from serine to alanine. The ConSurf sever gave a conservation score of 5 for this amino acid position, indicating semi-conservation. Two of the PredictSNP tools, namely SIFT and PANTHER, indicated that this amino acid substitution might affect *gyr*A activity, with an average accuracy of 73.4%. Using I-Mutant 3.0, this amino acid substitution was also predicted to decrease protein stability [DDG: − 0.8, reliability index (RI): 7]. There were no point mutations revealed in the DNA gyrase subunit B. Results are presented in Additional file 6. Furthermore, a gene encoding tetracycline resistance *tet*A was found in the genome of the HV carp isolate 04017018, displaying phenotypic resistance towards oxytetracycline.

To check for the presence of phages, the PHASTER tool identified 18 prophage sequences that were similar in the three newly assembled *F. columnare* strains (Additional file 7, Additional file 8A). These were all located on the same scaffold in a region of 12,451 bp. Some genes could be identified as subparts of the *Bacillus* phage G genome (PHAGE_Bacill_G_NC_023719). In the reference genome ATCC 49512, the latter prophage sequences were not identified. Instead, the PHASTER tool identified 37 prophage sequences identified as subparts of a *Flavobacterium* sp. (PHAGE_Flavob_23T_NC_041859) (Additional file 7, Additional file 8B). These were allocated on the same scaffold in a region of 50431 bp. However, as the scores of the prophages in the three newly assembled isolates and the reference strain were 30, respectively 40, and hence less than 70, the prophages seemed to be incomplete based on PHAST's completeness score calculation [44].

## Discussion

In the past years, more information has become available on the ability of, and stimulating factors for *F. columnare* to colonize surfaces and form biofilms [11, 15, 45, 46]. Bacterial motility is an important factor for the rapid colonization of a surface [46, 47]. Our former research conducted with the HV, LV and NV *F. columnare* isolates—also applied in this study—revealed that the HV *F. columnare* carp and trout isolates attached faster and in higher numbers to the gill tissue compared to the LV carp isolate [10, 11]. However, the latter also showed the capacity to rapidly colonize host tissue and cause tissue damage, followed by mortality of the fish. This was not the case for the NV trout isolate, which was not able to colonize tissues nor cause mortality [10, 11]. Therefore, the hypothesis arose that chemotaxis of the bacterial cells might play a crucial role in the colonization process. This hypothesis was reinforced based on the outcome of the SEED sequence based comparison analysis that was performed in this study. The software was used to differentiate protein-encoding genes present in the virulent isolates only and by comparing that generated output with the VFDB. A protein encoded gene annotated in RAST as a methyl-accepting chemotaxis protein (MCP) was present (Additional file 2). Extra analyses in the search for chemotaxis proteins via the VFDB in the four genomes revealed the presence of chemotaxis-involved genes *che*A, *che*B, *che*Y, and also *che*W-like proteins (results not shown). However, extra analyses with InterProScan in the search for signature sequences for chemotaxis such as pfam00015, or pfam00672 did not deliver further supportive evidence. This makes it difficult to state that chemotaxis is active in *F. columnare* based on currently identified genes and proteins. The importance of the *mcp*-gene is worth further investigation as chemotaxis has been described in the literature for *F. columnare* [13, 15] and this phenomenon must have a driving set of genes. If we could learn to understand and control chemotaxis processes, this might be an important step in the combat against columnaris disease. Comparing the effect of chemotaxis and colonization in wild type versus deletion mutants of this *mcp*-gene in the *F. columnare* isolates might be the first step in this puzzle.

The gliding motility in *F. columnare* is well-known [1]. The rate of motility has been linked to biofilm formation and the production of virulence factors in different pathogenic Gram-negative bacteria [47–49], including *F. columnare* [45, 46]. Prior to attachment, the bacterial cells are highly motile and glide over the surface. However, in this research, no differences were encountered between the four genomes in the secretion systems distribution involved in gliding. The few differences found in the other SS were not just observed only in the virulent or only in the non-virulent isolates. Hence these cannot explain possible differences in virulence. The mere presence or absence of these systems can hence probably not be attributed to virulence. This does not exclude that, based on environmental influence, a different expression pattern of the secretion systems and proteins involved in secretion can influence virulence or biofilm formation, as has been proven before for the isolates included in this study [45, 50].

As shown in Additional file 2, the identified virulence genes present in genomes of the virulent isolates but absent in the genome of the NV isolate, are related to asparagine synthetase and sugar transferases. In the human bacterial pathogen *Streptococcus pyogenes*, the expression of asparagine synthetase is important in the expression of streptolysin toxins. Sugar transferases, on the other hand, are important enzymes in establishing glycosidic linkages. As for *F. columnare*, sugars are known to play an important role in the adhesion of the bacteria to the host tissue [17]. It is no surprise that the genomes of the isolates that displayed virulence contain several unique sugar transferases compared to the genome of the NV trout isolate. The exact role of the asparagine synthetase and sugar transferases in the genome of the virulent isolates needs further exploration.

Further comparison of the genomes shows that the genome of the HV trout isolate possesses a unique gene encoding VgrG protein when compared to the NV trout isolate. Moreover, when comparing the genomes of the three virulent isolates versus the non-virulent one, the genomes of the virulent isolates possess two more unique genes encoding for the VgrG proteins (Additional file 2). VgrG is one of the key markers of the secretion apparatus of T6SS [51]. The T6SS is a versatile secretion system and might be involved in virulence, antagonism, nutrient acquisition, and horizontal gene transfer [52]. Contact‐dependent interbacterial competition appears to be the major function of T6SS, a function that can influence the composition of microbial communities [53]. While many bacterial pathogens, including *Pseudomonas aeruginosa*, *Vibrio cholerae*, and *Serratia marcescens*, are known to use their T6SSs under laboratory conditions [54–57], the mechanisms of how these T6SSs contribute to survival in the environment (and in host infection) have not been determined [54].

SEED Viewer allowed to identify another virulence-related gene encoding protein, a transcriptional regulator of the AraC family, in the genome of the HV carp isolate while absent in the one from the LV carp isolate (Additional file 3). Members of the AraC family of transcriptional regulators are thought to counteract histone-like nucleoid-structuring (H-NS)-induced silencing in select environments [58]. Under non-permissive metabolic and environmental conditions, H-NS represses gene expression by coating and/or condensing (bridging) DNA strands [59]. Depending on the bacterial growth phase, and in response to changes in environmental factors (e.g., temperature, osmolarity, and pH), H-NS will release DNA to enable gene expression [60–62]. In Enterobacteriaceae, the HNS protein contributes crucially to the fitness, including pathogenic and multidrug-resistant strains [60]. The latter makes sense for the HV carp isolate, resistant to oxytetracycline as determined before [25]. The AraC family protein in the HV carp isolate genome is flanked by a transposase, a putative prophage element, and a gene coding for TetA antimicrobial resistance protein (Figure 1).

A unique AraC-protein was also identified in the genome of the HV trout isolate as part of a genomic island NODE17 (Figure 2). Orthology analysis revealed that all genes in the latter region seem to be unique for the genome of the HV trout isolate when compared to the NV one, and encompasses at least three virulence gene-encoded proteins, namely AraC, NodT, and a LuxR family protein. The gene encoding AraC is flanked by genes encoding a putative mobilization protein and DNA topoisomerase III (Figure 2). As does AraC, the NodT protein could also play a role in antimicrobial resistance. NodT family proteins comprise a group of outer membrane lipoproteins of the Resistance-Nodulation-cell Division (RND) type efflux systems. These play a major role in both intrinsic and acquired multidrug resistance in Gram-negative bacteria [63]. Although no antimicrobial resistance had been found in the HV trout isolate in former research [25], the genome of this isolate has a series of genetic elements on board involving antimicrobial resistance mechanisms. However, these could be inactivated or silenced until reactivation. Apart from that, RND systems would also be important for the pathogenicity of several bacteria, e.g., *Salmonella enterica* [63]. The importance of these RND systems in both multidrug resistance and pathogenicity has made them a target of new drugs aimed at inhibiting their function [63] and might form a new interesting research field in *Flavobacterium* species as well. The third virulence-associated gene-encoded protein identified on NODE17 is the LuxR family protein, involved in quorum sensing (Figure 2). Currently, not much is known on how *F. columnare* cells communicate. Possibly indole, a sigal molecule identified in many pathogenic bacteria such as *E. coli*, several *Shigella* strains, *Enterococcus faecalis*, and *V. cholera* [64], might be involved. *F. columnare* is known to produce indole [1] and a gene encoded protein involved in indole-3-glycerol phosphate synthase was identified in the four *F. columnare* isolates studied in this manuscript (results not shown). Whether the latter has a signalling function, and what the role of the *lux*R-gene plays in QS of *F. columnare* remains unclear. Therefore, the way by which biofilm is regulated and formed in *F. columnare*, definitely deserves further research. Another remarkable feature in this part of the genome of the HV trout isolate is that the unique *lux*R family protein gene is flanked by *tra*-genes, the latter playing a crucial role in the transfer of genetic material, provided they are functional. NODE17 has been recognized as a genomic island by the IslandPaht-DIMOB and SIGI-HMM method. Losing (or acquiring) a genomic island may play a crucial role in the evolution of many bacteria, as it is involved in disseminating variable genes, including antibiotic resistance and virulence genes, and catabolic genes leading to the formation of new metabolic pathways [65]. The exact role in the genome of the HV *F. columnare* trout isolate remains to be investigated.

The four *F. columnare* isolates showed differences in the antimicrobial resistance profile based on minimum inhibitory concentration (MIC) values studied before [25]. This made it interesting to compare the four genomes in the search for underlying antimicrobial resistance mechanisms. Compared to the reference genome, not displaying any kind of phenotypic antimicrobial resistance, several single point mutations were discovered in four out of five gyrase genes present in *F. columnare* isolate CDI-A, the koi carp isolate displaying acquired resistance towards both first- and second-generation quinolones. Quinolone resistance would arise stepwise through selective amplification of mutants when drug concentrations are above the MIC-values of the susceptible population and below the MIC-values of the least susceptible mutant subpopulation [66]. Resistance towards quinolones due to mutations is frequently found in the quinolone-resistance-determining-region of the DNA gyrase subunit A (*gyr*A) [67]. Mutations in *gyr*A have been described to generate resistance to the first generation quinolones and reduced susceptibility to other quinolones [68]. Here, a unique SNP in the gyrase gene *gyr*A was found for isolate CDI-A, leading to an amino acid substitution. Software tools predicted that this substitution might affect gyrase activity and stability. This may indicate that this SNP is involved in decreased susceptibility of this isolate for fluoroquinolones. Nevertheless, further investigation is necessary, as these results do not necessarily imply a causal relationship between the presence of a SNP and antimicrobial resistance.

Furthermore, for the HV carp isolate 04017018, which displayed acquired phenotypic resistance towards oxytetracycline, a unique protein-encoding gene of interest was the tetracycline resistance gene *tet*A. Oxytetracycline is one of the most commonly used tetracyclines worldwide for the treatment of bacterial fish diseases [69]. Class A *tet* determinants can confer high-level tetracycline resistance [70], and have been found on transferable plasmids and in the chromosome of bacteria. In the genome of the HV carp isolate, *tet*A is flanked by a mobile element and a gene coding for chromosome (plasmid) partitioning protein *par*B (Figure 1), which is indicative of its mobile character. The PHASTER tool could identify several prophage sequences located on the same scaffold, but the scores of the prophage regions were less than 70, excluding the presence of a complete prophage in any of the four *F. columnare* isolates (Additional file 7). Hence, if *tet*A is positioned on a mobile element, it is not a prophage, according to these results. Further analyses are needed to investigate the mobile character of this gene.

Overall, a global analysis of the *F. columnare* genomes demonstrates that several unique genes involved in virulence are present in the genomes of the virulent *F. columnare* carp and trout isolates when compared to the non- or less virulent ones. The genomic analyses furthermore provide insights into possible acquired antimicrobial resistance mechanisms adopted by the bacterial cells. This study is the first to focus on identifying virulence genes based on the comparison between *F. columnare* isolates with different virulence and antimicrobial susceptibility patterns in carp and trout. Although much remains to be investigated with regard to mechanisms of e.g. chemotaxis, biofilm formation and quorum sensing, the findings in this study might form a basis for new genomic, virulence, and antimicrobial resistance studies.

## Supplementary Information

**Additional file 1.** **Comparison of** ***Flavobacterium columnare*** **strains.** (A) Amino acid composition for *F. columnare* strains. Letter abbreviations. A, Alanine; C, Cysteine; D, Aspartic Acid; E, Glutamic Acid; F, Phenylalanine; G, Glycine; H, Histidine; I, Isoleucine; K, Lysine; L, Leucine; M, Methionine; N, Asparagine; P, Proline; Q, Glutamine; R, Arginine; S, Serine; T, Threonine; V, Valine; W, Tryptophan; Y, Tyrosine. (B) Predicted protein lengths for *F. columnare* strains. (C) Predicted subcellular localization of proteins encoded in *F. columnare* strains using PSORTb. ATCC 49512: low virulent (LV) *F. columnare* trout isolate, JIP P11/91: highly virulent (HV) *F. columnare* trout isolate, CDI-A: LV *F. columnare* carp isolate, 04017018 : HV *F. columnare* carp isolate.

**Additional file 2.** **Protein-encoding genes present only in the virulent** ***F. columnare*** **genomes.** The latter 42 genes are only present in genomes of the highly virulent carp (04017018) and trout (JIPP11/91) and low virulent carp (CDI-A) *F. columnare* isolates that could cause tissue damage and mortality. These genes are not present in the genome of the non-virulent *F. columnare* trout isolate ATCC49512. Hypothetical genes (114) were not included here. Genes marked in bold are also predicted to be involved in virulence when identifying them via Virulence Factor Database.

**Additional file 3.** **Unique protein-encoding genes in the highly virulent carp isolate genome.** 80 genes were identified to be present in the genome of the highly virulent carp isolate 04017018 while absent in that of the low virulent carp isolate CDI-A. Of these 80 genes, the 62 hypothetical genes were left out in the representation above. Genes marked in bold are also predicted to be involved in virulence when identifying them via Virulence Factor Database. *Gene locations are formatted as NODEn:s-e where n is a unique scaffold number, s and e are the gene’s start and end position respectively.

**Additional file 4.** **Unique protein-encoding genes in the highly virulent trout isolate genome.** 374 genes were identified in the genome of the highly virulent *F. columnare* JIP P11/91 isolate while being absent in that of the non-virulent *F. columnare* ATCC 49512. Of these 374 genes, the 263 hypothetical genes were left out in the representation above. Genes marked in bold are also predicted to be involved in virulence when identifying them via Virulence Factor Database. *Gene locations are formatted as NODEn:s-e where n is a unique scaffold number, s and e are the gene’s start and end position respectively.

**Additional file 5.** **Single nucleotide variants (SNVs) in gyrase genes of** ***Flavobacterium columnare*** **isolates.** This table shows the SNVs present in the gyrase genes of *F. columnare* isolates CDI-A and 04017018 when compared to gyrase genes of the reference genome ATCC 49512. However, as only isolate CDI-A displayed phenotypic antimicrobial resistance towards both first- and second-generation quinolones, the unique SNVs in the gyrase genes of the latter isolate are highlighted in grey background. No SNVs were encountered in DNA gyrase subunit B (EC 5.99.1.3). Average read quality score of the bases supporting a variant was higher than 35 (which is considered very good quality).

**Additional file 6.** **Unique single nucleotide variants (SNVs) present in** ***gyr*A of isolate CDI-A.** The impact of the SNP (single nucleotide polymorphism) on protein activity/stability was predicted by comparison of amino acid characteristics, by using PredictSNP and I-Mutant Suite 3.0 tools, and by investigating which amino acids were present in the reference strain of *F. columnare* and of HV carp isolate 04017018, both of the latter not displaying acquired resistance towards fluoroquinolones, using BLAST and ConSurf.

**Additional file 7.** **Characteristics of prophage regions in the four** ***Flavobacterium columnare*** **isolates.** The prophage regions were identified in the genome of the reference isolate (ATCC49512) and the three newly assembled *F. columnare* isolates 04017018, CDI-A and JIP P11/91 via PHASTER. Prophage regions with scores less than 70 are incomplete.

**Additional file 8.** **Schematic representation of genes present in the prophages.** **A**: For the three newly sequenced bacterial genomes, the most probable prophage (Bacill_G_NC_023719) retrieved was similar for all three genomes. **B**: For the reference genome ATCC 49512, the latter prophage (Bacill_G_NC_023719) was not retrieved, however another most probable prophage (Flavob_23T_NC_041859) was identified. The prophages identified for all 4 isolates, were considered incomplete. Legend: Att: Attachment site, Coa: Coat protein (head protein), Hyp: Hypothetical proteins, PLP: Phage-like Protein, Tra: Transposase, Sha: Tail shaft.

## Data Availability

The genome sequence of isolate *Flavobacterium columnare* ATCC 49512 is publicly available from the NCBI database [26]. The sequencing reads and genomes of the 3 strains that were sequenced and assembled during this study are available in the NCBI-database under project PRJNA431138 [32] with the accession numbers as presented in Table 1 of this manuscript.

## Abbreviations

*F. columnare*: *Flavobacterium columnare*
*gld*: Gliding-gene
*gyr*A: DNA gyrase gene subunit A
HV: Highly virulent
LV: Low virulent
*mcp*: Methyl-accepting chemotaxis protein gene
MIC: Minimum Inhibitory Concentration
NV: Non-virulent
PanOCT: Pan-genome ortholog clustering tool
PHASTER: PHAge Search Tool-Enhanced Release
*por*: Porphyrin accumulation on the cell surface-gene
QS: Quorum sensing
RND: Resistance Nodulation Cell Division
SNP: Single nucleotide polymorphism
*spr*: Spreading-gene
T4SS: Type IV Secretion system
T6SS: Type VI Secretion system
*tet*A: Tetracycline A gene
VFDB: Virulence factor database
