# Supplementary figures and images for: Comparative genomics of Flavobacterium columnare unveils novel insights in virulence and antimicrobial resistance mechanisms
Source: Vet Res. 2021 Feb 12;52:18. doi: 10.1186/s13567-021-00899-w (PMC7881675; doi:10.1186/s13567-021-00899-w)

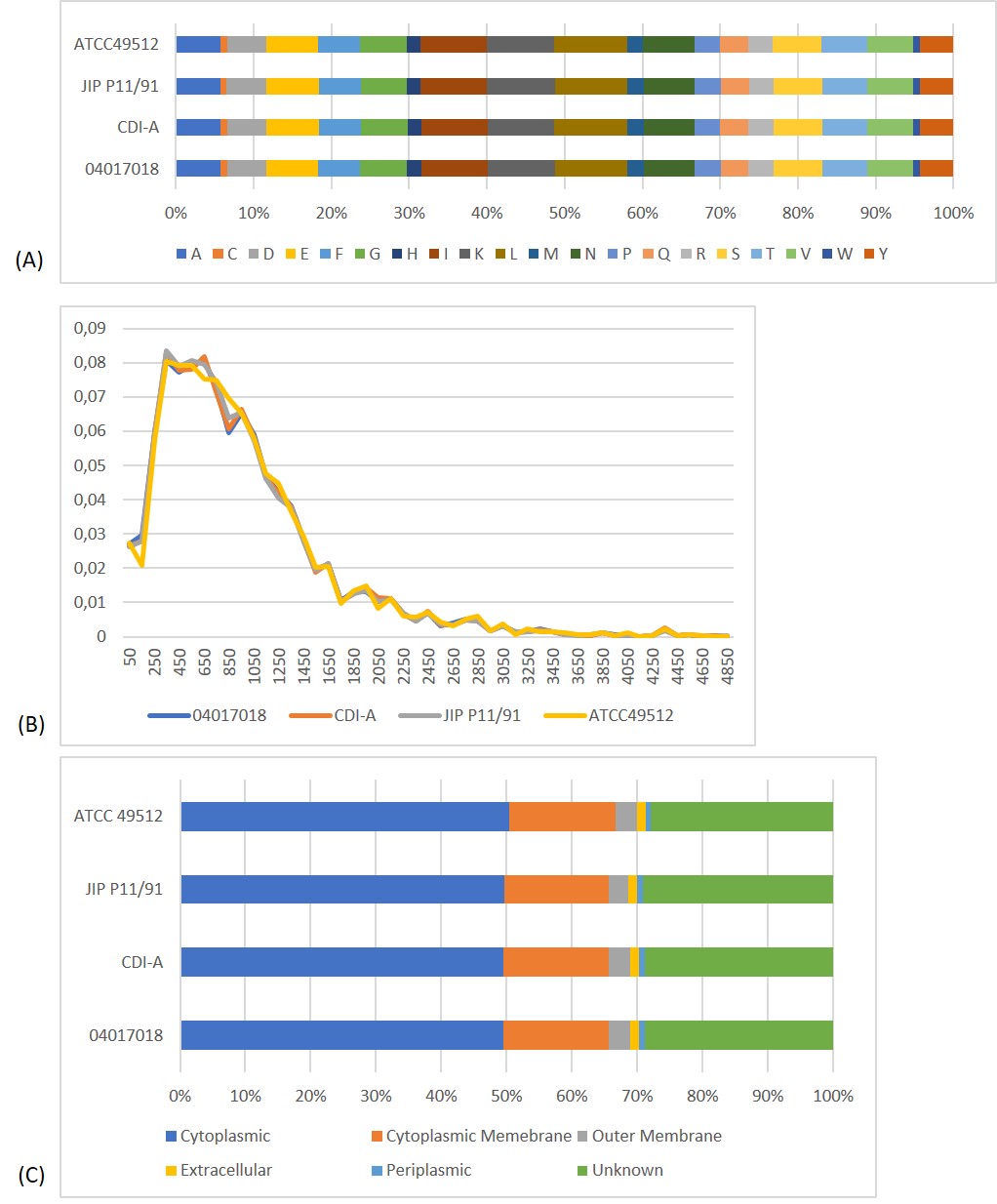

Supplement: Supplementary file 1 — Additional file 1. Comparison of Flavobacterium columnare strains. (A) Amino acid composition for F. columnare strains. Letter abbreviations. A, Alanine; C, Cysteine; D, Aspartic Acid; E, Glutamic Acid; F, Phenylalanine; G, Glycine; H, Histidine; I, Isoleucine; K, Lysine; L, Leucine; M, Methionine; N, Asparagine; P, Proline; Q, Glutamine; R, Arginine; S, Serine; T, Threonine; V, Valine; W, Tryptophan; Y, Tyrosine. (B) Predicted protein lengths for F. columnare strains. (C) Predicted subcellular localization of proteins encoded in F. columnare strains using PSORTb. ATCC 49512: low virulent (LV) F. columnare trout isolate, JIP P11/91: highly virulent (HV) F. columnare trout isolate, CDI-A: LV F. columnare carp isolate, 04017018 : HV F. columnare carp isolate. [file 13567_2021_899_MOESM1_ESM.jpg]

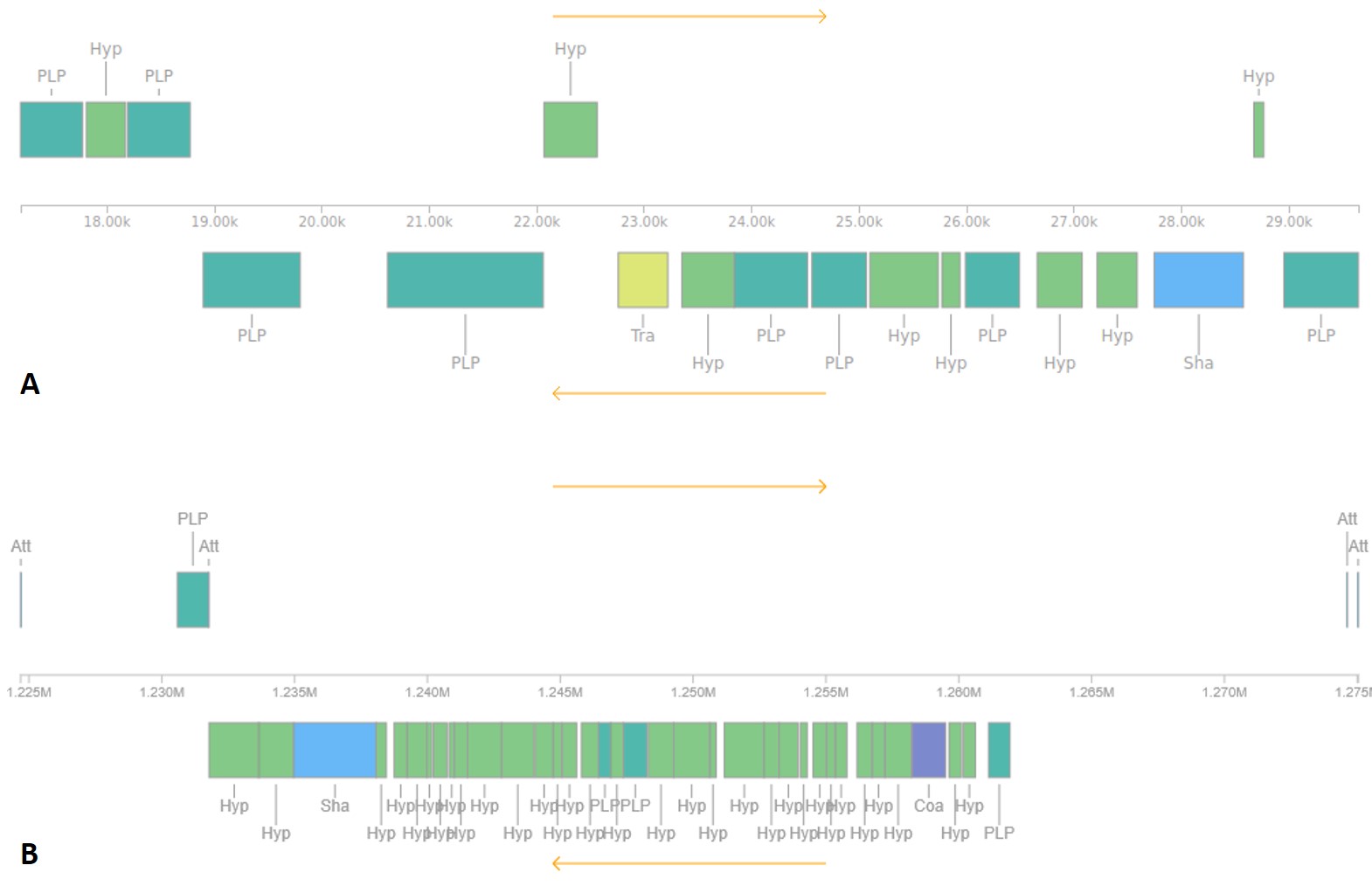

Supplement: Supplementary file 8 — Additional file 8. Schematic representation of genes present in the prophages. A: For the three newly sequenced bacterial genomes, the most probable prophage (Bacill_G_NC_023719) retrieved was similar for all three genomes. B: For the reference genome ATCC 49512, the latter prophage (Bacill_G_NC_023719) was not retrieved, however another most probable prophage (Flavob_23T_NC_041859) was identified. The prophages identified for all 4 isolates, were considered incomplete. Legend: Att: Attachment site, Coa: Coat protein (head protein), Hyp: Hypothetical proteins, PLP: Phage-like Protein, Tra: Transposase, Sha: Tail shaft. [file 13567_2021_899_MOESM8_ESM.jpg]
