# Supplementary material for: Comparative genomics of Flavobacterium columnare unveils novel insights in virulence and antimicrobial resistance mechanisms
Source: Vet Res. 2021 Feb 12;52:18. doi: 10.1186/s13567-021-00899-w (PMC7881675; doi:10.1186/s13567-021-00899-w)
Supplement: Supplementary file 2 — Additional file 2. Protein-encoding genes present only in the virulent F. columnare genomes. The latter 42 genes are only present in genomes of the highly virulent carp (04017018) and trout (JIPP11/91) and low virulent carp (CDI-A) F. columnare isolates that could cause tissue damage and mortality. These genes are not present in the genome of the non-virulent F. columnare trout isolate ATCC49512. Hypothetical genes (114) were not included here. Genes marked in bold are also predicted to be involved in virulence when identifying them via Virulence Factor Database. [file 13567_2021_899_MOESM2_ESM.docx]

**Additional file 2: Protein-encoding genes present only in the virulent *F. columnare* genomes.**

| **Function** | **Gene location in 04017018** | **Gene location in CDI-A** | **Gene location in JIPP11/91** |
| --- | --- | --- | --- |
| 4-amino-6-deoxy-N-Acetyl-D-hexosaminyl-(Lipid carrier) acetyltransferase | NODE14:30295-30912 | NODE17:30682-31299 | NODE11:30295-30912 |
| Adenine-specific methyltransferase | NODE7:77294-80188 | NODE5:complement(54811-57705) | NODE72:336-3230 |
| **Alpha-1,3-N-acetylgalactosamine transferase PglA (EC 2.4.1.-)** | NODE14:28637-29710 | NODE17:29024-30097 | NODE11:28637-29710 |
| **Asparagine synthetase [glutamine-hydrolyzing] (EC 6.3.5.4)** | NODE14:23339-25225 | NODE17:23726-25612 | NODE11:23339-25225 |
| ATP/GTP-binding protein | NODE27:complement(37637-38965) | NODE27:complement(37647-38975) | NODE96:278-1606 |
| ATPase involved in DNA repair | NODE16:32018-33826 | NODE21:complement(22849-24657) | NODE26:18986-20737 |
| Cartilage oligometric matrix protein precursor (COMP) | NODE43:complement(5105-5932) | NODE42:6745-7098 | NODE53:complement(1675-2028) |
| Conserved domain protein | NODE3:complement(99164-100195) | NODE3:49870-50901 | NODE5:45305-46336 |
| CRISPR-associated protein Cas2 | NODE23:36771-37076 | NODE25:complement(13642-13971) | NODE30:complement(1880-2185) |
| DNA double-strand break repair protein Mre11 | NODE1:complement(8704-10026) | NODE7:117827-119149 | NODE9:87459-88781 |
| DNA double-strand break repair Rad50 ATPase | NODE1:complement(4759-8061) | NODE7:119792-123094 | NODE9:89550-92726 |
| DNA-cytosine methyltransferase (EC 2.1.1.37) | NODE27:7189-8445 | NODE27:7183-8445 | NODE31:complement(28587-29849) |
| **glycosyl transferase, family 2** | NODE14:58904-60448 | NODE17:59291-60835 | NODE11:58905-60449 |
| Integrase | NODE1:94043-95272 | NODE7:complement(32581-33810) | NODE74:377-1606 |
| Lipid carrier : UDP-N-acetylgalactosaminyltransferase (EC 2.4.1.-) | NODE14:29727-30305 | NODE17:30114-30692 | NODE11:29727-30305 |
| Lipopolysaccharide biosynthesis protein RffA | NODE14:30905-32032 | NODE17:31292-32419 | NODE11:30905-32032 |
| Lipopolysaccharide modification acyltransferase | NODE14:52286-53359 | NODE17:52673-53746 | NODE11:52286-53359 |
| Membrane protein | NODE14:22163-23335 | NODE17:22610-23722 | NODE11:22223-23335 |
| **Membrane protein involved in the export of O-antigen, teichoic acid lipoteichoic acids** | NODE14:20819-22153 | NODE17:21206-22540 | NODE11:20819-22153 |
| **Methyl-accepting chemotaxis protein** | NODE3:complement(151647-152120) | NODE44:514-987 | NODE54:668-1161 |
| **pathogenesis related protein** | NODE2:complement(93980-96037) | NODE1:63904-65961 | NODE3:63448-65505 |
| Peptidoglycan-binding domain 1 | NODE2:complement(156932-158584) | NODE1:1388-3040 | NODE3:922-2574 |
| Phage/plasmid primase P4, C-terminal | NODE1:96357-97757 | NODE7:complement(30096-31496) | NODE74:2691-4091 |
| Polysaccharide deacetylase | NODE14:26302-27258 | NODE17:26689-27645 | NODE11:26302-27258 |
| Possible DNA helicase | NODE2:complement(92054-93976) | NODE1:65965-67887 | NODE3:65509-67431 |
| **Predicted ATP-binding protein involved in virulence** | NODE4:complement(141022-143322) | NODE29:complement(36025-38325) | NODE86:66-446 |
| Probable transposase | NODE24:complement(22758-23219) | NODE23:complement(24154-24615) | NODE4:complement(22758-23024) |
| Putative helicase | NODE1:complement(105795-108950) | NODE7:18903-22058 | NODE69:complement(311-3466) |
| Putative toxin component near putative ESAT-related proteins, repetitive / Repetitive hypothetical protein near ESAT cluster, SA0282 homolog | NODE5:31658-32743 | NODE4:complement(107486-108529) | NODE33:complement(1495-2532) |
| Putative transcriptional regulatory protein | NODE44:3840-4193 | NODE42:6745-7098 | NODE53:complement(1675-2028) |
| RloF | NODE4:130445-132121 | NODE29:25469-27145 | NODE75:709-2385 |
| **Sugar transferase** | NODE14:51423-52283 | NODE17:51810-52670 | NODE11:51423-52283 |
| Tetracycline efflux protein TetA | NODE42:complement(7062-8381) | NODE58:108-1043 | NODE82:complement(277-1596) |
| Transcriptional regulator | NODE6:complement(22516-22755) | NODE6:111804-112043 | NODE73:792-1031 |
| Transcriptional regulator | NODE48:complement(532-867) | NODE54:complement(1770-2105) | NODE79:1503-1838 |
| Type I restriction-modification system, DNA-methyltransferase subunit M (EC 2.1.1.72) | NODE1:complement(101753-103987) | NODE7:23866-26100 | NODE67:232-2466 |
| Type I restriction-modification system, DNA-methyltransferase subunit M (EC 2.1.1.72) | NODE6:19702-21414 | NODE6:complement(113145-114857) | NODE73:complement(2133-3845) |
| Type I restriction-modification system, specificity subunit S (EC 3.1.21.3) | NODE1:complement(100396-101751) | NODE7:26102-27457 | NODE67:2468-3823 |
| Type II restriction endonuclease, putative | NODE6:complement(21401-22519) | NODE6:112040-113158 | NODE73:1028-2146 |
| Ulcer associated adenine specific DNA methyltransferase | NODE4:complement(4743-5852) | NODE13:99383-100492 | NODE29:35946-37055 |
| **VgrG protein** | NODE1:complement(182317-182685) | NODE22:93-461 | NODE47:complement(12486-12854) |
| **VgrG protein** | NODE39:120-482 | NODE19:complement(76616-76978) | NODE43:complement(18457-18819) |

The latter 42 genes are only present in the highly virulent carp (04017018) and trout (JIPP11/91) and low virulent carp (CDI-A) *F. columnare* isolates that could cause tissue damage and mortality. These genes are not present in the non-virulent *F. columnare* trout isolate ATCC49512. Hypothetical genes (114) were not included here. Genes marked in bold are also predicted to be involved in virulence when identifying them via Virulence Factor Database
