# Supplementary material for: Comparative genomics of Flavobacterium columnare unveils novel insights in virulence and antimicrobial resistance mechanisms
Source: Vet Res. 2021 Feb 12;52:18. doi: 10.1186/s13567-021-00899-w (PMC7881675; doi:10.1186/s13567-021-00899-w)
Supplement: Supplementary file 3 — Additional file 3. Unique protein-encoding genes in the highly virulent carp isolate genome. 80 genes were identified to be present in the genome of the highly virulent carp isolate 04017018 while absent in that of the low virulent carp isolate CDI-A. Of these 80 genes, the 62 hypothetical genes were left out in the representation above. Genes marked in bold are also predicted to be involved in virulence when identifying them via Virulence Factor Database. *Gene locations are formatted as NODEn:s-e where n is a unique scaffold number, s and e are the gene’s start and end position respectively. [file 13567_2021_899_MOESM3_ESM.docx]

**Additional file 3. Unique protein-encoding genes in the highly virulent carp isolate genome.**

| **Function** | **Gene location* in 04017018** |
| --- | --- |
| Type I restriction-modification system, specificity subunit S (EC 3.1.21.3) | NODE3:144578-146236 |
| Type I restriction-modification system, DNA-methyltransferase subunit M (EC 2.1.1.72) | NODE3:148798-150261 |
| Gll0036 protein | NODE3:complement(1949-2529) |
| Endoribonuclease L-PSP | NODE3:complement(2533-2925) |
| transcriptional regulator, DeoR family s | NODE3:complement(3012-3704) |
| Conserved membrane-spanning protein | NODE3:complement(3771-4499) |
| Putative prophage protein (ps3) | NODE42:complement(5046-5627) |
| Chromosome (plasmid) partitioning protein ParB / Stage 0 sporulation protein J | NODE42:complement(8431-9441) |
| Mobile element protein | NODE42:complement(9462-9815) |
| Tyrosine type site-specific recombinase | NODE8:112097-113299 |
| Putative bacteriophage protein | NODE8:115966-116826 |
| Nucleoid-associated protein NdpA | NODE8:complement(119006-120004) |
| DNA repair protein RadC | NODE8:120777-121217 |
| TPR repeat | NODE8:122880-123590 |
| Mobile element protein | NODE38:21275-21709 |
| **Transcriptional regulator, AraC family** | NODE42:complement(3102-3539) |
| **Glycosyltransferase involved in LPS biosynthesis** | NODE6:complement(123808-124008) |
| Rhodanese-like domain protein | NODE6:complement(126900-127220) |

80 genes were identified to be present in the genome of the highly virulent carp isolate 04017018 while absent in that of the low virulent carp isolate CDI-A. Of these 80 genes, the 62 hypothetical genes were left out in the representation above. Genes marked in bold are also predicted to be involved in virulence when identifying them via Virulence Factor Database. *Gene locations are formatted as NODEn:s-e where n is a unique scaffold number, s and e are the gene’s start and end position respectively.
