# Supplementary material for: Comparative genomics of Flavobacterium columnare unveils novel insights in virulence and antimicrobial resistance mechanisms
Source: Vet Res. 2021 Feb 12;52:18. doi: 10.1186/s13567-021-00899-w (PMC7881675; doi:10.1186/s13567-021-00899-w)
Supplement: Supplementary file 4 — Additional file 4. Unique protein-encoding genes in the highly virulent trout isolate genome. 374 genes were identified in the genome of the highly virulent F. columnare JIP P11/91 isolate while being absent in that of the non-virulent F. columnare ATCC 49512. Of these 374 genes, the 263 hypothetical genes were left out in the representation above. Genes marked in bold are also predicted to be involved in virulence when identifying them via Virulence Factor Database. *Gene locations are formatted as NODEn:s-e where n is a unique scaffold number, s and e are the gene’s start and end position respectively. [file 13567_2021_899_MOESM4_ESM.docx]

**Additional file 4. Unique protein-encoding genes in the highly virulent trout isolate genome**

| **Function** | **Gene location* in JIPP11/91** |
| --- | --- |
| 4-amino-6-deoxy-N-Acetyl-D-hexosaminyl-(Lipid carrier) acetyltransferase | NODE11:30295-30912 |
| Adenine-specific methyltransferase | NODE72:336-3230 |
| Alpha-1,3-N-acetylgalactosamine transferase PglA (EC 2.4.1.-) | NODE11:28637-29710 |
| ATP/GTP-binding protein | NODE96:278-1606 |
| ATPase involved in DNA repair | NODE26:18986-20737 |
| ATP-dependent DNA helicase pcrA (EC 3.6.1.-) | NODE17:24120-26051 |
| Azurin | NODE71:718-1206 |
| BNR repeat domain protein | NODE10:67624-79182 |
| Carboxynorspermidine dehydrogenase | NODE17:complement(51158-52390) |
| Cartilage oligomeric matrix protein precursor (COMP) | NODE53:6657-7484 |
| Cartilage oligomeric matrix protein precursor (COMP) | NODE80:complement(926-1768) |
| CDS_ID OB0245 | NODE17:complement(54175-54867) |
| Chromosome (plasmid) partitioning protein ParB / Stage 0 sporulation protein J | NODE82:complement(1646-2656) |
| Conjugative transposon protein TraA | NODE17:complement(27182-28147) |
| Conjugative transposon protein TraB | NODE17:complement(26728-27177) |
| Conjugative transposon protein TraD | NODE17:complement(26081-26587) |
| Conjugative transposon protein TraE | NODE17:complement(21229-21534) |
| Conjugative transposon protein TraG | NODE17:complement(18384-20888) |
| Conjugative transposon protein TraI | NODE17:complement(17724-18356) |
| Conjugative transposon protein TraJ | NODE17:complement(16725-17720) |
| Conjugative transposon protein TraK | NODE17: complement(16081-16704) |
| Conjugative transposon protein TraM | NODE17:complement(14010-15347) |
| Conjugative transposon protein TraN | NODE17:complement(13094-13987) |
| Conjugative transposon protein TraO | NODE17:complement(12513-13073) |
| Conjugative transposon protein TraQ | NODE17:complement(12052-12501) |
| Conserved domain protein | NODE5:45305-46336 |
| CRISPR-associated protein Cas2 | NODE30:complement(1880-2185) |
| DNA double-strand break repair protein Mre11 | NODE9:87459-88781 |
| DNA double-strand break repair Rad50 ATPase | NODE9:89550-92726 |
| DNA primase (EC 2.7.7.-) | NODE56:complement(62-2392) |
| DNA primase (EC 2.7.7.-) | NODE65:complement(4907-5578) |
| DNA repair protein RadC | NODE83:complement(2901-3341) |
| DNA topoisomerase III (EC 5.99.1.2) | NODE17:complement(44551-46635) |
| DNA-binding protein | NODE7:53680-54144 |
| **DNA-binding response regulator, LuxR family** | NODE17:complement(8305-8706) |
| DNA-cytosine methyltransferase (EC 2.1.1.37) | NODE31:complement(28587-29849) |
| efflux transporter, RND family, MFP subunit | NODE17:complement(59570-60466) |
| Endonuclease V (EC 3.1.21.7) | NODE8:1398-2681 |
| Esterase | NODE17:35763-36716 |
| Ethidium bromide-methyl viologen resistance protein EmrE | NODE71:1865-2194 |
| Filamentation induced by cAMP protein Fic | NODE7:54334-54720 |
| glycosyl transferase, family 2 | NODE11:58905-60449 |
| helix-turn-helix domain protein | NODE17:21739-22038 |
| HNH endocuclease | NODE15:complement(68567-69643) |
| Integrase | NODE17:382-1707 |
| Integrase | NODE74:377-1606 |
| Lipid carrier : UDP-N-acetylgalactosaminyltransferase (EC 2.4.1.-) | NODE11:29727-30305 |
| Lipopolysaccharide biosynthesis protein RffA | NODE11:30905-32032 |
| Lipopolysaccharide modification acyltransferase | NODE11:52286-53359 |
| **Membrane protein** | NODE11:22223-23335 |
| **Membrane protein involved in the export of O-antigen, teichoic acid lipoteichoic acids** | NODE11:20819-22153 |
| **Methyl-accepting chemotaxis protein** | NODE54:688-1161 |
| Methyl-directed repair DNA adenine methylase (EC 2.1.1.72) | NODE17:30860-31771 |
| Mobile element protein | NODE82:complement(2677-3030) |
| Modification methylase EcoRI (EC 2.1.1.72) | NODE15:complement(69640-70632) |
| Mycobacteriophage Barnyard protein gp56 | NODE17:2604-3677 |
| NADPH:quinone oxidoreductase 2 ## possible protective/detoxification role | NODE17:complement(50262-51131) |
| Nucleoid-associated protein NdpA | NODE52:3147-4214 |
| Nucleoid-associated protein NdpA | NODE90:759-1757 |
| Pathogenesis related protein | NODE17:22057-24138 |
| Pathogenesis related protein | NODE3:63448-65505 |
| Peptidase, family M23 (EC 3.4.24.-) | NODE58:273-1511 |
| Peptidoglycan-binding domain 1 | NODE3:922-2574 |
| Phage/plasmid primase P4, C-terminal | NODE74:2691-4091 |
| Polysaccharide deacetylase | NODE11:26302-27258 |
| possible DNA helicase | NODE3:65509-67431 |
| Predicted ATP-binding protein involved in virulence | NODE86:66-446 |
| Predicted Zn peptidase | NODE54:5945-6739 |
| Probable endonuclease | NODE14:complement(7514-8272) |
| Probable transposase | NODE4:complement(22758-23024) |
| Putative bacteriophage protein | NODE88: complement(1220-2080) |
| Putative conjugative transposon mobilization protein BF0132 | NODE17:29142-30425 |
| Putative DNA methylase | NODE17:complement(37731-43139) |
| Putative helicase | NODE69:complement(311-3466) |
| Putative mobilization protein BF0133 | NODE17:33634-35628 |
| Putative prophage protein (ps3) | NODE92:1286-1867 |
| Putative toxin component near putative ESAT-related proteins, repetitive / Repetitive hypothetical protein near ESAT cluster, SA0282 homolog | NODE33:complement(1495-2532) |
| Putative transcriptional regulatory protein | NODE53:complement(1675-2028) |
| Related to MCBG protein | NODE71:2656-3243 |
| Ribosomal protein S1 | NODE65:complement(2266-3159) |
| RloF | NODE75:709-2385 |
| RND efflux system, inner membrane transporter CmeB | NODE17:complement(56402-59545) |
| **RND efflux system, outer membrane lipoprotein, NodT family** | NODE17:complement(54957-56384) |
| Single-stranded DNA-binding protein | NODE17:2168-2560 |
| Site-specific tyrosine recombinase | NODE102:complement(18-914) |
| **Sugar transferase** | NODE11:51423-52283 |
| Tetracycline efflux protein TetA | NODE82:complement(277-1596) |
| Tetracycline resistance element mobilization regulatory protein rteC | NODE17:complement(49142-49987) |
| Tetracycline resistance element mobilization regulatory protein rteC | NODE71:3372-4208 |
| ThiF family protein, ubiquitin-activating enzyme | NODE17:7266-8072 |
| Topoisomerase IV subunit B (EC 5.99.1.-) | NODE31:105-236 |
| Topoisomerase IV subunit B (EC 5.99.1.-) | NODE96:105-236 |
| TPR repeat | NODE83:complement(528-1238) |
| Transcriptional regulator | NODE73:792-1031 |
| Transcriptional regulator | NODE79:1503-1838 |
| **Transcriptional regulator, AraC family** | NODE17:36752-37645 |
| Transcriptional regulator, TetR family | NODE71:1302-1868 |
| Transcriptional repressor of PBSX genes | NODE10:complement(87739-88074) |
| Type I restriction-modification system, DNA-methyltransferase subunit M (EC 2.1.1.72) | NODE67:232-2466 |
| Type I restriction-modification system, DNA-methyltransferase subunit M (EC 2.1.1.72) | NODE73:complement(2133-3845) |
| Type I restriction-modification system, specificity subunit S (EC 3.1.21.3) | NODE67:2468-3823 |
| Type I restriction-modification system, specificity subunit S (EC 3.1.21.3) | NODE81:246-1904 |
| Type II restriction endonuclease, putative | NODE73:1028-2146 |
| Type IIs restriction endonuclease | NODE17:31777-33456 |
| Tyrosine recombinase XerC | NODE102:complement(968-1888) |
| Tyrosine type site-specific recombinase | NODE99:complement(982-2184) |
| Ulcer associated adenine specific DNA methyltransferase | NODE29:35946-37055 |
| Universal stress protein family | NODE17:complement(52463-52900) |
| **VgrG protein** | **NODE43:complement(18457-18819)** |
| **VgrG protein** | **NODE47:complement(5031-5630)** |
| **VgrG protein** | **NODE47:complement(12486-12854)** |

374 genes were identified in the genome of the highly virulent *F. columnare* JIP P11/91 isolate while being absent in that of the non-virulent *F. columnare* ATCC 49512. Of these 374 genes, the 263 hypothetical genes were left out in the representation above. Genes marked in bold are also predicted to be involved in virulence when identifying them via Virulence Factor Database. *Gene locations are formatted as NODEn:s-e where n is a unique scaffold number, s and e are the gene’s start and end position respectively.
