# Supplementary material for: Comparative genomics of Flavobacterium columnare unveils novel insights in virulence and antimicrobial resistance mechanisms
Source: Vet Res. 2021 Feb 12;52:18. doi: 10.1186/s13567-021-00899-w (PMC7881675; doi:10.1186/s13567-021-00899-w)
Supplement: Supplementary file 5 — Additional file 5. Single nucleotide variants (SNVs) in gyrase genes of Flavobacterium columnare isolates. This table shows the SNVs present in the gyrase genes of F. columnare isolates CDI-A and 04017018 when compared to gyrase genes of the reference genome ATCC 49512. However, as only isolate CDI-A displayed phenotypic antimicrobial resistance towards both first- and second-generation quinolones, the unique SNVs in the gyrase genes of the latter isolate are highlighted in grey background. No SNVs were encountered in DNA gyrase subunit B (EC 5.99.1.3). Average read quality score of the bases supporting a variant was higher than 35 (which is considered very good quality). [file 13567_2021_899_MOESM5_ESM.docx]

**Additional file 5** **Single nucleotide variants (SNVs) in gyrase genes of *F. columnare* isolates**

| **Chromosome** | **Region** | **Type** | **Reference** | **Allele** | **Reference allele** | **Zygosity** | **Count** | **Coverage** | **Frequency** | **Forward read count** | **Reverse read count** | **Forward/reverse balance** |
| --- | --- | --- | --- | --- | --- | --- | --- | --- | --- | --- | --- | --- |
| **NODE2:complement(3752-6310)**  **DNA gyrase subunit A (EC 5.99.1.3)** | 244 | SNV | T | G | No | Homozygous | 191 | 191 | 100 | 137 | 83 | 0.377273 |
| NODE27:complement(32411-35116)  Topoisomerase IV subunit A (EC 5.99.1.-) | 1221 | SNV | G | T | No | Homozygous | 121 | 121 | 100 | 79 | 58 | 0.423358 |
| NODE27:complement(32411-35116)  Topoisomerase IV subunit A (EC 5.99.1.-) | 2067 | SNV | T | C | No | Homozygous | 139 | 139 | 100 | 75 | 79 | 0.487013 |
| NODE27:complement(39017-40879)  Topoisomerase IV subunit B (EC 5.99.1.-) | 154 | SNV | G | A | No | Homozygous | 174 | 174 | 100 | 142 | 61 | 0.300493 |
| NODE27:complement(39017-40879)  Topoisomerase IV subunit B (EC 5.99.1.-) | 1779 | SNV | T | G | No | Homozygous | 49 | 49 | 100 | 10 | 49 | 0.169492 |
| NODE27:complement(39017-40879)  Topoisomerase IV subunit B (EC 5.99.1.-) | 1782 | SNV | A | G | No | Homozygous | 35 | 35 | 100 | 8 | 35 | 0.186047 |
| **NODE15:74090-76591**  **DNA topoisomerase I (EC 5.99.1.2)** | 273 | SNV | A | G | No | Homozygous | 144 | 144 | 100 | 101 | 61 | 0.376543 |
| **NODE15:74090-76591DNA topoisomerase I (EC 5.99.1.2)** | 405 | SNV | G | A | No | Homozygous | 175 | 175 | 100 | 106 | 105 | 0.49763 |
| **NODE15:74090-76591DNA topoisomerase I (EC 5.99.1.2)** | 468 | SNV | G | A | No | Homozygous | 183 | 183 | 100 | 128 | 77 | 0.37561 |
| NODE15:74090-76591DNA topoisomerase I (EC 5.99.1.2) | 617 | SNV | G | A | No | Homozygous | 218 | 219 | 99,54338 | 97 | 147 | 0.397541 |
| NODE15:74090-76591DNA topoisomerase I (EC 5.99.1.2) | 2151 | SNV | A | G | No | Homozygous | 178 | 178 | 100 | 104 | 104 | 0.5 |

This table shows the SNVs present in the gyrase genes of *F. columnare* isolates CDI-A and 04017018 when comparing it to gyrase genes of the reference genome ATCC 49512. However, as only isolate CDI-A displayed phenotypic antimicrobial resistance towards both first- and second-generation quinolones, the unique SNV in the gyrase genes of the latter isolate are marked in bold. No SNVs were encountered in DNA gyrase subunit B (EC 5.99.1.3). Average read quality score of the bases supporting a variant was higher than 35 (which is considered very good quality).
