# Supplementary material for: Comparative genomics of Flavobacterium columnare unveils novel insights in virulence and antimicrobial resistance mechanisms
Source: Vet Res. 2021 Feb 12;52:18. doi: 10.1186/s13567-021-00899-w (PMC7881675; doi:10.1186/s13567-021-00899-w)
Supplement: Supplementary file 6 — Additional file 6. Unique single nucleotide variants (SNVs) present in gyrA of isolate CDI-A. The impact of the SNP (single nucleotide polymorphism) on protein activity/stability was predicted by comparison of amino acid characteristics, by using PredictSNP and I-Mutant Suite 3.0 tools, and by investigating which amino acids were present in the reference strain of F. columnare and of HV carp isolate 04017018, both of the latter not displaying acquired resistance towards fluoroquinolones, using BLAST and ConSurf. [file 13567_2021_899_MOESM6_ESM.docx]

**Additional file 6: Unique single nucleotide variants (SNVs) present in *gyr*A of isolate CDI-A**

| **Location** | | **Codon and corresponding amino acid** | | **Impact SNPs on protein activity/stability** | | | |
| --- | --- | --- | --- | --- | --- | --- | --- |
| **Gene** | **SNP-position** | **non-wild type isolate(s)** | **wild type isolate(s)** | **amino acid characteristics** | **predictSNP (accuracy %)** | **I-Mutant 3.0** | **ConSurf** |
| *gyrase* A *(gyr*A*)* | 244 | Alanine (A) | Serine (S) | A: apolar  S: polar amino acid | deleterious (73.4%) | DDG: -0.8, RI: 7 | other species: various amino acids, CS: 5 |

The impact of the SNP (single nucleotide polymorphism) on protein activity/stability was predicted by comparison of amino acid characteristics, by using PredictSNP and I-Mutant Suite 3.0 tools, and by investigating which amino acids were present in the reference strain of *F. columnare* and of HV carp isolate 04017018, both of the latter not displaying acquired resistance towards fluoroquinolones, using BLAST and ConSurf.
