# Supplementary material for: Comparative genomics of Flavobacterium columnare unveils novel insights in virulence and antimicrobial resistance mechanisms
Source: Vet Res. 2021 Feb 12;52:18. doi: 10.1186/s13567-021-00899-w (PMC7881675; doi:10.1186/s13567-021-00899-w)
Supplement: Supplementary file 7 — Additional file 7. Characteristics of prophage regions in the four Flavobacterium columnare isolates. The prophage regions were identified in the genome of the reference isolate (ATCC49512) and the three newly assembled F. columnare isolates 04017018, CDI-A and JIP P11/91 via PHASTER. Prophage regions with scores less than 70 are incomplete. [file 13567_2021_899_MOESM7_ESM.docx]

**Additional file 7: Characteristics of prophage regions in the four *Flavobacterium columnare* isolates**

| Sample | Length (Kb) | Score | Total Proteins | Phage hit proteins | Region Position | GC % | Most Common Phage |
| --- | --- | --- | --- | --- | --- | --- | --- |
| ATCC49512 | 50.3 | 40 | 37 | 15 | [1224688-1275028](https://phaster.ca/submissions/ZZ_5a5cd36a10#region_dna0) | 33.13 | PHAGE_Flavob_23T_NC_041859(7) |
| 04017018 | 12.4 | 30 | 18 | 13 | NODE24:17196-29646 | 34.27 | PHAGE_Bacill_G_NC_023719(4) |
| CDI-A | 12.4 | 30 | 18 | 13 | NODE23:18592-31042 | 34.27 | PHAGE_Bacill_G_NC_023719(4) |
| JIP P11/91 | 12.4 | 30 | 18 | 13 | NODE4:17196-29646 | 34.29 | PHAGE_Bacill_G_NC_023719(4) |

The prophage regions were identified in the reference isolate (ATCC49512) and the three newly assembled *F. columnare* isolates 04017018, CDI-A and JIP P11/91 via PHASTER. Prophage regions with scores less than 70 are incomplete
